# Serological Evidence of Immune Priming by Group A Streptococci in Patients with Acute Rheumatic Fever

**DOI:** 10.3389/fmicb.2016.01119

**Published:** 2016-07-22

**Authors:** Jeremy M. Raynes, Hannah R. C. Frost, Deborah A. Williamson, Paul G. Young, Edward N. Baker, John D. Steemson, Jacelyn M. Loh, Thomas Proft, P. R. Dunbar, Polly E. Atatoa Carr, Anita Bell, Nicole J. Moreland

**Affiliations:** ^1^School of Biological Sciences, University of AucklandAuckland, New Zealand; ^2^Maurice Wilkins Centre for Molecular Biodiscovery, University of AucklandAuckland, New Zealand; ^3^Institute of Environmental Science and ResearchWellington, New Zealand; ^4^The Peter Doherty Institute, University of MelbourneMelbourne, Australia; ^5^School of Medical Sciences, University of AucklandAuckland, New Zealand; ^6^Waikato District Health BoardHamilton, New Zealand

**Keywords:** acute rheumatic fever, T-antigen, group A *Streptococcus*, tee-type, immune priming, Immunohistochemistry

## Abstract

Acute rheumatic fever (ARF) is an autoimmune response to Group A *Streptococcus* (GAS) infection. Repeated GAS exposures are proposed to ‘prime’ the immune system for autoimmunity. This notion of immune-priming by multiple GAS infections was first postulated in the 1960s, but direct experimental evidence to support the hypothesis has been lacking. Here, we present novel methodology, based on antibody responses to GAS T-antigens, that enables previous GAS exposures to be mapped in patient sera. T-antigens are surface expressed, type specific antigens and GAS strains fall into 18 major clades or T-types. A panel of recombinant T-antigens was generated and immunoassays were performed in parallel with serum depletion experiments allowing type-specific T-antigen antibodies to be distinguished from cross-reactive antibodies. At least two distinct GAS exposures were detected in each of the ARF sera tested. Furthermore, no two sera had the same T-antigen reactivity profile suggesting that each patient was exposed to a unique series of GAS T-types prior to developing ARF. The methods have provided much-needed experimental evidence to substantiate the immune-priming hypothesis, and will facilitate further serological profiling studies that explore the multifaceted interactions between GAS and the host.

## Introduction

Acute rheumatic fever (ARF) is an autoimmune condition that can develop after a Group A *Streptococcus* (GAS) infection. ARF is now rare in high-income countries, but is associated with significant disease burden in low-income countries and some indigenous populations of high-income countries (Carapetis et al., 2016). The rates of ARF in Māori and Pacific children in New Zealand and Aboriginal children in Australia are amongst the highest in the world (Jaine et al., 2008; Maguire et al., 2012). The peak incidence for ARF occurs in the 5–14 years old age band, with a mean peak in 9–12 year olds observed in a recent study (Jaine et al., 2008). Episodes in children younger than 5 years of age are extremely rare. It has been postulated that repeated infections with GAS are needed to ‘prime’ the immune system before the first episode of ARF occurs (Carapetis et al., 2005, 2016). This may partly explain the lack of disease in pre-school children. Superficial GAS infections (pharyngitis and impetigo) are regularly reported in the under 5′s (Steer et al., 2007; Shaikh et al., 2010; Romani et al., 2015), and if multiple exposures to GAS are needed to trigger autoimmune symptoms this would contribute to the peak age for ARF being in older children.

Direct experimental evidence to support the notion of ‘immune priming’ by repeated infections is lacking, most likely because a prospective study designed to follow GAS infections in children prior to development of ARF would be extremely difficult to conduct. Even in areas of high disease burden, patient numbers would need to be large and the cohort retained over many years. Rather the current ‘immune priming’ hypothesis is based almost entirely on indirect evidence obtained from examining the cross-reactive immune response in ARF (Carapetis et al., 2016). The notion that repeated GAS infections were needed to trigger autoimmunity was first postulated by Zabriskie (1967). Zabriskie described a single case of recurrent ARF, where two GAS infections had occurred in the 8 years between the child’s first and second hospital admission for ARF. Based on this observation Zabriskie speculated that repeated GAS episodes were necessary for disease to occur. This is certainly a logical argument that fits with the current understandings of disease pathogenesis. Shared epitopes between coiled-coil GAS proteins and human heart proteins (molecular mimicry) are thought to be important targets for cross-reactive antibodies and T-cells in ARF (Cunningham, 2014). Indeed, ARF patients have elevated antibody titres to selected GAS antigens and human heart proteins compared with healthy controls (Cunningham et al., 1989; Martins et al., 2006; Ellis et al., 2010). It follows that repeated GAS infections would be required to generate molecular mimicry, a loss of tolerance and eventually autoimmunity. However, none of the studies examining antibody titres in ARF patients determined the frequency of previous GAS infections. Rather they measured total GAS antibody titres in sera and these antibodies could have been generated during any number of previous GAS exposures. In order to systematically explore the basis of immune priming, new approaches are needed to accurately determine the frequency of GAS exposures in children with ARF.

## A Serological Approach to Examine Immune Priming

The spectrum of antibodies in human sera is dynamic and is shaped by previous encounters with infectious agents. In effect it is a ‘molecular record’ of pathological insults and by characterizing antibody specificities in polyclonal serum it is possible to map these prior insults or exposures (Weiss-Ottolenghi and Gershoni, 2014). The entire repertoire of antibodies in sera has been coined the ‘IgOme’ and several laboratories are now developing high-throughput methods to define specific components of the IgOme or ‘serum memory’ in high resolution (Weiss-Ottolenghi and Gershoni, 2014; Xu et al., 2015). For example, a recent ground-breaking study comprehensively profiled previous viral exposures in serum samples from hundreds of human volunteers using peptide sequences derived from all known human viruses (Xu et al., 2015). Mapping the breadth and frequency of pathological insults associated with immune-mediated, infectious diseases such as ARF is crucial to advancing our understanding of disease pathogenesis. The success of such a mapping approach is reliant on identifying GAS peptides or proteins that generate persistent, type-specific antibodies during infection.

The two major type-specific antigens expressed by GAS, and historically used in serotyping techniques developed by Lancefield, are the M protein and T-antigen (Cunningham, 2000). Today GAS is most commonly typed by sequencing the 5′ end of the *emm* gene, which encodes the M protein (Beall et al., 1996), with over 200 *emm-*types identified to date (Steer et al., 2009). The T-antigen, and associated *tee-*typing is a supplementary typing tool sometimes used in epidemiological assessments (Luca-Harari et al., 2009). The T-antigen shows significantly less antigenic variation than the M protein, with a recent survey of GAS isolates in New Zealand finding that *tee* genes fall into 18 major clades or *tee-*types (Steemson et al., 2014). This reduced variation, combined with previous evidence that T-antigens are immunogenic and expressed during the course of human infection (Manetti et al., 2007; Young et al., 2014), makes the T-antigen an ideal tool to examine the spectrum of GAS exposures in children with ARF.

## Building a T-Antigen Protein Array

To develop methods to explore the immune priming hypothesis, sera were obtained from seven patients of Māori ethnicity, diagnosed with first-episode ARF according to the New Zealand modification of the Jones criteria (Atatoa-Carr et al., 2008; **Table 1**). Two of the participants (S1 and S2) were siblings. Written informed consent was obtained from all patients, and ethical approval was provided from the New Zealand Central Regional Ethics Committee. Throat swabs were performed on all patients at admission and GAS isolates were obtained from two patients. These isolates were *tee-*typed using published protocols (Steemson et al., 2014) and determined to be *tee3.2* (patient A4) and *tee13* (patient S2). The *tee* genes were cloned and the corresponding T-antigens (T3.2 and T13) were produced recombinantly in *Escherichia coli* as described for other T-antigens (Kang et al., 2007; Young et al., 2014). A further 12 T-antigens were also produced using standard techniques (**Table 1**). Our previous work has shown that the *tee* genes fall into 18 major clades. The 14 T-antigens included in our panel represent the major *tee-*types currently circulating in New Zealand (Steemson et al., 2014).

**Table 1:** Serum IgG endpoint titre of rheumatic fever patient sera against a panel of 14 recombinant Group A *Streptococcus* (GAS) T-antigens.

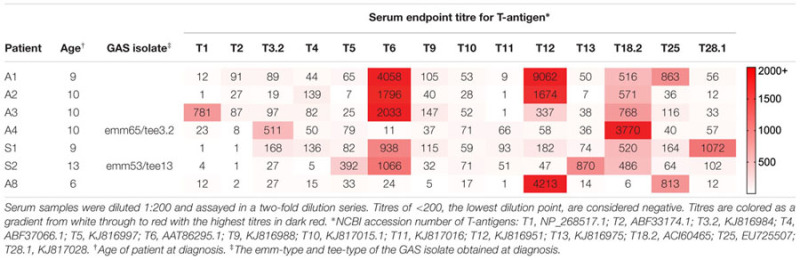

ELISAs performed with the 14 recombinant T-antigens, patient sera and an anti-human IgG secondary antibody show that all ARF patients have elevated antibody titres to two or more T-antigens (**Table 1**). Each patient has a distinct T-antigen reactivity profile with no two sera recognizing the same set of T-antigens. Some T-antigens (T6, T12, T18.2, and T25) are recognized by multiple patient sera, while other T-antigens (T1, T3.2, T5, T13, and T28.1) are recognized by a single patient. The T-antigens cloned from the GAS isolates obtained at diagnosis from patient A4 (T3.2) and S2 (T13) are recognized by their respective patient serum.

Of note, the two participants that were siblings (S1 and S2) had distinct differences in their T-antigen reactivity profiles. Sibling S1 serum does not recognize T13, the T-antigen expressed by the isolate obtained from sibling S2 at hospital admission. The siblings were admitted to hospital within 3 days of each other, attended the same school and lived together. Yet the results suggest the siblings were infected with GAS strains of different *tee*-types immediately prior to developing ARF.

## Mapping Gas Exposures Using T-Antigens

The reactivity of patient serum to multiple T-antigens observed in this study has also been observed in previous studies, albeit with much smaller panels of T-antigens (Manetti et al., 2007; Young et al., 2014). It suggests an individual has experienced repeated infections with GAS strains carrying different T-antigens, or that cross-reactivity exists amongst homologous T-antigen epitopes, or a combination of the two. In order to dissect these possibilities serum depletion experiments were performed with T-antigen affinity columns. Columns were generated by coupling T-antigens to beaded agarose using an AminoLink Immobilization Kit (Thermo Scientific).

**FIGURE 1:**
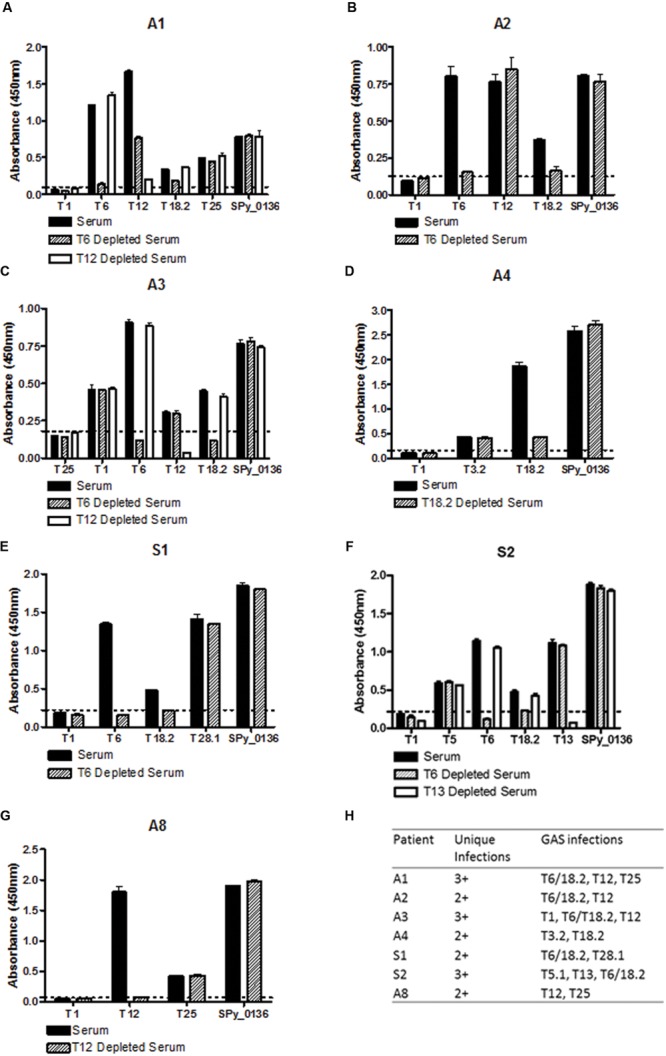
**Mapping Group A *Streptococcus* (GAS) infection history using a T-antigen array.** Sera were obtained from seven patients diagnosed with first-episode Acute rheumatic fever (ARF). For depletion of T-antigen specific antibodies sera were diluted 1:100 in PBS pH 7.4 and passed over a T-antigen column. Unbound antibodies (depleted serum) were collected for analysis by ELISA. T-antigens (5 μg/mL) were coated onto immunoplates, incubated with patient serum (1 h at 37**°**C) and total IgG binding detected. Reactivity of un-depleted serum (solid bars) and depleted serum (crosshatched and white bars) for each participant is shown in **A–G**, and a summary of the data is shown in **H**. All depletion ELISAs included a non-reactive T-antigen as a negative control and the conserved GAS protein Spy_0136 (NP_268523) as a non-T-antigen positive control. Samples were considered to have a specific T-antigen antibody response if the absorbance values were at least two standard deviations higher than the mean of the negative control (dotted line). The depletion process did not affect serum reactivity with the positive control.

The depletion data show that most of the T-antigen reactivity observed is type-specific (**Figure 1**) – when sera are passed over a T-antigen column the antibodies that react with that particular T-antigen are removed but reactivity to other T-antigens remains unchanged. For example, when patient A2 serum is depleted for T6 antibodies the reactivity for T12 remains unchanged (**Figure 1B**) indicating the T6 and T12 antibodies originate from unique infections. Cross-reactivity does occur but this is limited to specific T-types. For all patients with T6 reactivity (patients A1, A2, A3, S1, and S2), depletion of T6 antibodies also removed T18.2 antibodies demonstrating shared epitopes on T6 and T18.2. A single patient (A1) generated T6 specific antibodies that partially cross-react with T12, indicated by the reduction, but not complete removal of T12 antibodies, following T6 antibody depletion (**Figure 1A**).

Additional depletion experiments were performed for patients in whom the number of T-antigen exposures could not be determined from a single depletion. For example, for sibling S2 the reactivity to T13 and T5.1 was unchanged by T6 depletion whereas reactivity to T6 and T5.1 was unchanged by T13 depletion (**Figure 1F**). This shows that sibling S2 has T-antigen antibodies from three distinct GAS infections (T13, T5.1, and T6/T18.2). Taken together, the depletion assays show that all seven patients have T-antigen antibodies from at least two distinct GAS infections, and three of the seven patients have antibodies from three distinct infections (**Figure 1H**).

## Discussion

This perspective describes methodology that enables immunological determination of GAS exposures in patient sera. A recombinant panel of T-antigens that represent GAS *tee-*types currently circulating in New Zealand was generated. Utilizing immunoassays in parallel with serum depletion experiments enabled type-specific T-antigen antibodies to be distinguished from cross-reactive antibodies. This, in turn, enabled the number of GAS exposures with strains of different *tee-*type to be quantified in sera. When applied to ARF, all of the patients tested had at least two separate GAS exposures prior to developing the disease. To the best of our knowledge this is the first experimental evidence that ‘immune priming’ with repeated GAS infections occurs prior to the development of first-episode ARF.

The methodology is based on the premise that exposure to infectious agents such as bacteria and viruses leaves a ‘footprint’ on the immune system. Humoral responses to infection are normally detected within 10–14 days of an encounter and can persist for years, or even decades, after an exposure. The idea that serum provides a ‘molecular record’ of prior pathological insults has provided the basis for innovative new research that profiles serum antibodies at unprecedented resolution (the so called IgOme) (Weiss-Ottolenghi and Gershoni, 2014; Xu et al., 2015). The persistence of GAS serum antibodies for many years after infection was first demonstrated by Lancefield (1959). This, combined with previous data published by our laboratory and others showing that T-antigens elicit a strong antibody response during human infection (Manetti et al., 2007; Young et al., 2014), provided the necessary evidence that serum profiling by T-type was a tractable methodology for GAS.

Heterogeneity was observed across the ARF patient group with respect to the *tee-*types each patient had encountered. No two patients had the same T-antigen reactivity profile suggesting every patient was exposed to a unique series of GAS *tee*-types prior to developing ARF. These observed differences, in particular for the concurrently diagnosed siblings (patient S1 and S2), highlight the complex relationship between GAS infection and the development of ARF. This adds to the ongoing debate around whether certain GAS strains are ‘rheumatogenic,’ or, as these data might suggest, the development of ARF is not driven by a specific GAS strain but rather the dysregulation of the host immune response following infection. This is supported by our recent analysis of GAS isolates associated with ARF in New Zealand in which a lack of classical ‘rheumatogenic’ strains was observed and a diverse range of *emm-*types were associated with disease (Williamson et al., 2015).

There are several limitations to the methodology described. GAS cluster into 18 major *tee*-types and the 14 major *tee*-types currently circulating in New Zealand are included in the current analysis (Steemson et al., 2014). It is possible that additional GAS exposures might have been detected if the remaining four *tee-*types were included in the synthetic array, although the lower prevalence of these *tee*-types in New Zealand makes its unlikely that their inclusion would significantly alter the study findings. The current methodology is unable to distinguish between repeated infections with the same *tee-*type. Additional type-specific antigens would be needed to distinguish between infections of the same *tee*-type. While this may enable additional exposures to be detected it would also add a level of complexity to the current methodology. The main rationale for selecting T-type rather than M-type to map prior exposures was tractability. The reduced number of T-antigens required to cover the major circulating *tee-*types enabled type-specific and cross-reactive antibodies to be dissected with ease using serum depletion methodology. In contrast an M-protein array would need to be significantly larger to cover the major circulating *emm-*types. Over 220 *emm*-types have been identified to date (Steer et al., 2009), and at a minimum a synthetic array would need to encompass representatives from the 48 major *emm-*clusters identified in a recent functional classification of M-proteins (Sanderson-Smith et al., 2014). Furthermore, dissection of type-specific and cross-reactive M protein responses would be challenging given the heptad repeat motifs that span M protein sequences (Smeesters et al., 2010).

## Concluding Remarks

The methods described enable immunological determination of GAS exposures in patient sera by T*-*type. Effectiveness has been demonstrated in seven children with ARF by showing that each child was exposed to GAS at least twice prior to developing disease. This has provided much-needed experimental evidence to substantiate the ‘immune priming’ hypothesis postulated by Zabriskie some 50 years ago, which stated that repeated GAS episodes were necessary for ARF to occur.

## Author Contributions

NM, JR, DW, PAC, and AB conceived the study. NM, JR, PD, EB, and TP designed the experiments. NM, JR, HF, JS, JL, and PY performed the experiments. NM, PAC, and AB recruited the patients and all authors contributed to writing the manuscript.

## Conflict of Interest Statement

The authors declare that the research was conducted in the absence of any commercial or financial relationships that could be construed as a potential conflict of interest.
